# A pilot study on bacterial isolates associated with purulent vaginal discharge in dairy cows in the south‐west region of Western Australia

**DOI:** 10.1111/avj.13152

**Published:** 2022-03-03

**Authors:** PA Ludbey, S Sahibzada, CH Annandale, ID Robertson, FK Waichigo, MS Tufail, JL Valenzuela, JW Aleri

**Affiliations:** ^1^ School of Veterinary Medicine, Murdoch University Murdoch Western Australia 6150 Australia; ^2^ Antimicrobial Resistance and Infectious Diseases Research Laboratory Murdoch University Murdoch Western Australia 6150 Australia; ^3^ College of Veterinary Medicine, Huazhong Agricultural University Wuhan Hubei 430070 China; ^4^ Brunswick Veterinary Services Brunswick Junction Western Australia 6224 Australia; ^5^ Centre for Animal Production and Health Future Foods Institute, Murdoch University Murdoch Western Australia 6150 Australia

**Keywords:** antimicrobial resistance, bacteria, dairy cattle, endometritis

## Abstract

This study aimed to determine the bacterial isolates associated with postpartum endometritis among dairy cows in Western Australia and their antimicrobial susceptibility profiles. A cross‐sectional study was conducted between June–October 2020. Endometritis was defined as evidence of mucopurulent to purulent vaginal discharge 60–100 days postpartum. Vaginal discharge samples were obtained, cultured, identified and tested for antimicrobial susceptibility. A total of 118 bacterial isolates were grown from 46 animals, representing 36 species. The bacteria isolated from both aerobic and anaerobic cultures included *Bacillus* (60.2%), *Streptococcus* (12.7%), *Trueperella* (10.1%), *Escherichia* (6.7%) and *Staphylococcus* (5.9%). The remaining genera <5% were *Histophilus*, *Aeroccocus*, *Enterococcus* and *Moraxella*. Resistance was variable between isolates, but the highest resistance levels were observed in *Streptococcal* and *Bacillus* isolates to enrofloxacin, clindamycin and erythromycin, respectively. All *Streptococcal* isolates exhibited 100% resistance to enrofloxacin, and the greatest resistance levels were found in *Streptococcus luteinises* to trimethoprim‐sulfamethoxazole 83%, clindamycin 66% and 33% quinupristin‐dalfopristin. There was 84.5% resistance to clindamycin and 35.2% to erythromycin in the *Bacillus* isolates, with the highest resistance found in *Bacillus licheniformis* and *Bacillus subtilis*. *Escherichia coli* exhibited 12.5% resistance to gentamycin, ceftiofur, whereas amoxicillin‐clavulanic acid exhibited 37.5%. Within the *Staphylococcal* isolates, 28.5%, 28.5%, 42.8% and 14.2% resistance to ceftiofur, erythromycin, cefoxitin, penicillin and tetracycline were observed, respectively. The presence of resistance to important antimicrobials for human use, such as cephalosporins, macrolides and fluoroquinolones, highlights the need for judicious use of antimicrobials in dairy cattle.

Endometritis a form of purulent vaginal discharge is characterised by persistent bacterial infection of the uterus, delaying return to oestrus in the postpartum period in 15%–40% of dairy cows and subsequent infertility.^1^, ^2^, ^3^ Substantial economic losses can occur in the dairy industry as a result of infection of the uterus from the cost of treatment, reduced fertility, and cost of replacing culled animals.^4^, ^5^, ^6^ Common treatment options for purulent vaginal discharge centre around the use of prostaglandin analogues and antimicrobials.^7^, ^8^, ^9^ Antimicrobial use in the treatment of purulent vaginal discharge has come into question partly because of the growing prevalence of antimicrobial resistance (AMR).^10^, ^11^, ^12^ AMR can occur with any antimicrobial use, and it's more likely to occur with overuse of broad‐spectrum antimicrobials and low dosing or inappropriate dosage length.^10^, ^13^, ^14^ Spontaneous recovery has also been documented,^15^, ^16^ and post‐treatment improvement infertility is variable. The aetiological agents causing purulent vaginal discharge in dairy cattle include *Escherichia coli*, *Trueperella pyogenes*, *Fusobacterium necrophorum* and *Prevotella melaninogenicus*.^17^, ^18^, ^19^, ^20^ Although these bacterial pathogens are commonly associated with endometritis, no specific combination of organisms has been consistently identified. Many other bacteria, including *Streptococcus* spp., *Staphylococcus* spp., *Bacteroides* spp., *Bacillus* spp. and *Clostridial* spp., have also been isolated.^19^, ^20^, ^21^ Understanding the bacterial species found in purulent vaginal discharge and their antimicrobial susceptibility profiles is necessary to establish appropriate antimicrobial stewardship practices to slow the AMR rate. This study endeavours to determine the bacterial pathogens and susceptibility profiles present in cases of purulent vaginal discharge found in Western Australian dairy herds.

## Materials and methods

### Study approval, location and design

This is a cross‐sectional study of six farms conducted in dairy herds located in the south‐west region of Western Australia between June–October 2020. Farms were selected based on herd availability. This study was approved by the animal ethics committee at Murdoch University (permit no. R3238/20).

### Study populations, selection of study farms and animals

Sixty‐four animals from six farms were selected for this study from 125 Holstein Friesian dairy cows at 60–100 days postpartum, based on nonpregnancy and evidence of mucopurulent to purulent vaginal discharge. This window criterion was deemed reasonable considering a year‐round calving pattern and 80 days submission rate as an important reproductive index. The sampled animals were from six farming properties, all with pasture‐based production systems. A vaginal discharge score (VDS) was assigned to each animal, following the 0–3 scale system established by Williams et al.^22^ This system defines as VDS 0 animals with no mucus or clear mucus, VDS 1 animals with a discharge containing flecks of white or off‐white pus, VDS 2 animals with discharge containing less than 50% white or off‐white mucopurulent pus and VDS 3 animals with discharge containing more than 50% white or yellow purulent pus.

### General data collection

Each animal was sampled using a sterile Metricheck device (Simcro, Hamilton, New Zealand).^23^ The perineal area was gently scrubbed using a 7.5% Iodine scrub (Vetsense PVP‐iodine scrub, Mulgrave, NSW, Aust) and water before drying with a paper towel. After that, the perineal area was disinfected with 70% Isopropyl alcohol. Each sample was placed into a labelled sterile collection pot.

### Sample processing

Laboratory investigation was undertaken at Murdoch University. A 10 μL sample of endometrial fluid was pipetted on to plates containing media of Muller‐Hinton Agar (MHA) + 5% Sheep Blood (SB) and streaked using a sterile 10 μL inoculating loop. This process was repeated twice for each sample to obtain both aerobic and anaerobic cultures. Anaerobic samples were placed in an airtight jar with Anaerogen™ sachets (Oxoid™). Both sample types (aerobic and anaerobic) were then placed in an incubator at 37°C for 24 h.

### Identification of colonies

Colonies were identified according to their morphological characteristics, growth pattern, shape, colour, and haemolytic properties. Further, the identified individual colonies were isolated and re‐cultured using a 1 μL sterile inoculating loop on MHA + 5% SB media plates. In rare cases, where initial bacteriology revealed no growth, the samples were re‐cultured again. A sample was assigned as negative if there was no growth after two rounds of incubation (each for 24 h). The obtained fresh pure colonies were run through Matrix Assisted Laser Desorption Ionization Time of Flight Mass Spectrometry (MALDI‐TOF) for species identification using manufacturing methods. Bacterial isolates with a MALDI‐TOF score ≥1.80 were interpreted as a reliable identification and thus used for further antimicrobial susceptibility testing, while isolates with a score <1.8 were considered unreliable and hence excluded from further testing.

### Antimicrobial susceptibility testing

Once the pure colonies were isolated and identified, a disk diffusion antibiotic test was performed using either MHA or MHA + 5% SB in the case of *streptococci*. A single pure colony was picked with a sterile 1 μL loop and added into sterile 0.9% sodium chloride solution. The mixture was then resuspended via hand mixing and vortexing to reach a turbidity of the 0.5 McFarland Turbidity Standard.^24^ A sterile cotton tip was used to inoculate MHA plates. For fastidious organisms, 5% Sheep Blood Mueller‐Hinton agar and chocolate agar plates were used. The antibiotic disks were then applied with the help of a disk dispenser, and the plates were then incubated at 37°C for 24 h under either anaerobic or aerobic conditions.

Antimicrobial susceptibility was tested for 12 antibiotics; amoxicillin‐clavulanate 10/20 μg (AMC), ceftiofur 30 μg (CER), cefoxitin 30 μg (FOX), clindamycin 2 μg (CLI), chloramphenicol 30 μg (CHL), erythromycin 15 μg (ERY), enrofloxacin 5 μg (ENR), gentamicin 10 μg (GEN), penicillin 10 μg (PEN), quinupristin‐dalfopristin 15 μg (QD), tetracycline 30 μg (TET) and trimethoprim‐sulfamethoxazole 25 μg (SXT). Diffusion disks were purchased from Thermo Scientific™ (Oxoid™, Massachusetts, USA).

Disk zone diameters were read after 24 h of incubation using digital callipers. The diameter across each antibiotic disk was measured and recorded. Bacterial growth inhibition was then evaluated, and results were categorised as resistant or susceptible. Antimicrobial susceptibility results were interpreted using Clinical and Laboratory Standards Institute (CLSI) guidelines.^25^ All intermediate resistance isolates were considered susceptible for prevalence estimation. A total of 36 different species were isolated and tested against 12 commonly used antimicrobials. Of these, three species *T*. *pyogenes*, *Moraxella bovis* and *Aeroccocus viridians* had no standards for comparison or were intrinsically resistant to all the antimicrobials tested. As such, a table depicting the zone diameters for each is included (see Appendix Tables A1 and B1).

## Results

### General descriptions

Samples were obtained from six farms. The total number of cows sampled on each farm was as follows; farm 1 n = 3, farm 2 n = 5, farm 3 n = 9, farm 4 n = 1, farm 5 n = 25 and farm 6 n = 24. A total of 67 cows with variable vaginal discharge scores (VDS) were sampled. Of these, 46 cows had growth, and 21 had negative growth. VDS was recorded for each animal and ranged from 0 to 3.^22^ Of all isolates, 42.8% came from animals with a VDS of 0, 22.6% with a VDS of 1, 6.7% VDS 2 and 27.7% from VDS 3 (Table 1).

**Table 1:** Vaginal discharge scores collected from dairy cows with endometritis and percentage of isolated bacteria derived from each score

	Vaginal discharge score							
	Score 0		Score 1		Score 2		Score 3	
% of total cows sampled	42.80%		22.60%		6.70%		27.70%	
	n = 51		n = 27		n = 8		n = 33	
Bacterial species	No	%	No.	%	No.	%	No.	%
*Bacillus* spp.	42	82%	13	48%	5	62.5%	11	33.3%
*Escherichia* spp.	1	2%	3	11%	0	0%	4	12.1%
*Staphylococcal* spp.	3	5.8%	3	11%	0	0%	1	3.00%
*Streptococcal* spp.	3	5.8%	7	26%	3	37.5%	4	12.1%
*Trueperella* spp.	0	0%	0	0%	0	0%	12	36.3%
*Histophilus* spp.	1	2%	1	3.7%	0	0%	0	0%
*Aeroccocus* spp.	1	2%	0	0%	0	0%	0	0%
*Enterococcus* spp.	0	0%	0	0%	0	0%	1	3%

N, number of cows sampled from each vaginal discharge score; no., number of isolates within each genus; %, percentage of each genus isolated from each vaginal discharge score.

### Bacterial isolates

A total of 118 bacterial isolates were grown from the 46 positive samples with more than one isolate in each sample, representing 36 different microorganisms. The main microorganisms isolated in this study were *Bacillus* (60.2%; 71/118), *Streptococcus* (12.7%, 15/118), *Trueperella* (10.1%, 12/118), *Escherichia* (6.7%, 8/118) and *Staphylococcus* (5.9%, 7/118). The remaining making up <5% were *Histophilus*, *Aeroccocus*, *Enterococcus* and *Moraxella*. *Bacillus* spp. was composed of 18 different species. The most predominant were *B*. *licheniformis* 42.8% (30/70), *B*. *subtilus* 12.8% (9/70), *Bacillus altitudinis* 7.1% (5/70), *Bacillus borstenlensis* 7.1% (5/70) and *Bacillus sonorensis* 5.7% (4/70) the rest were a mix of 11 species making up 24.2% (17/70). *Streptococcus* was the next common species isolated and was composed *Streptococcus luteinises* 40% (6/15), *Streptococcus pluranimalium* 33.3% (5/15) and *Streptococcus uberis* 13.3% (2/15) and singular isolates of *Streptococcus alactolyticus* 6.6% (1/15) and *Streptococcus equinus* 6.6% (1/15). The *Staphylococcus* species was composed of *Staphylococcus microti*, *Staphylococcus warneri*, *Staphylococcus equorum*, *Staphylococcus hyicus*, *Staphylococcus hominis*, *Staphylococcus kloosi* and *Staphylococcus chromogenes*, with each species being isolated once. *T*. *pyogenes* was isolated 12 times, and *E*. *coli* 8 times. Three additional species were isolated in low abundance and included *Histophilus somni*, *Aeroccocus viridans*, *M*. *bovis* and *Enterococcus hirae*.

### Antimicrobial susceptibility

*Streptococcus* was composed of five species (Table 2), with all isolates 100% (15/15) resistant to ENR, 60% (9/15) SXT, 46.6% (7/15) CLI, 13.3% (2/15) resistant to QD, 6% resistant to TET and ERY. No resistance was found to AMC, CER, CHL or PEN. At an individual species level (Table 2) the greatest resistance levels were found in *S*. *luteinises*, which was isolated 6 times with resistance found in all six to ENR 100% (6/6), SXT 83% (5/6), CLI 66% (4/6) and 33% (2/6) to QD. On a species level (Table 3), 85% (60/70) of the *Bacillus* isolates were resistant to CLI, and 35% (25/70) resistance was found to ERY.

**Table 2:** Percentage of resistance to 12 antimicrobials within *Streptococcal* species isolated from dairy cattle with endometritis

*Streptococcus* spp.	*S*. *Pluranimalium*		*S*. *Lutetiensis*		*S*. *Uberis*		*S*. *equinus*		*S*.*alactolyticus*	
	Overall (n = 5)		Overall (n = 6)		Overall (n = 2)		Overall (n = 1)		Overall (n = 1)	
Antimicrobial	No.	R%	No.	R%	No.	R%	No.	R%	No.	R%
Amoxicillin‐clavulanate	0	0%	0	0%	0	0%	0	0%	0	0%
Ceftiofur	0	0%	0	0%	0	0%	0	0%	0	0%
Chloramphenicol	0	0%	0	0%	0	0%	0	0%	0	0%
Clindamycin	0	0%	4	66.60%	0	0%	1	0%	1	100%
Enrofloxacin	5	100%	6	100%	2	100%	1	100%	1	100%
Erythromycin	0	0%	0	0%	0	0%	0	0%	0	0%
Cefoxitin	‐	‐	‐	‐	‐	‐	‐	‐	‐	‐
Gentamicin	‐	‐	‐	‐	‐	‐	‐	‐	‐	‐
Penicillin	0	0%	0	0%	0	0%	0	0%	0	0%
Quinupristin‐ dalfopristin	0	0%	2	33.30%	0	0%	0	0%	0	0%
Trimethoprim‐sulfamethoxazole	1	20%	5	83.30%	1	50%	0	0%	1	100%
Tetracycline	0	0%	0	0%	0	0%	0	0%	0	0%

R%, percentage of resistance within each species; n, sample size of each species; No., number of resistant isolates within each species.

**Table 3:** Total number of resistant isolates per genus from dairy cattle with endometritis

Genus	AMC	CER	CHL	CLI	ENR	ERY	FOX	GEN	PEN	QD	SXT	TET
*Bacillus*	0	0	0	60	0	25	0	0	0	0	0	0
*Enterococcus*	0	0	0	1	0	0	0	0	0	0	0	0
*Escherichia*	3	1	0	0	0	0	1	1	0	0	0	0
*Histophilus*	0	0	0	0	0	0	0	0	0	0	0	0
*Staphylococcus*	0	2	0	0	0	2	3	0	3	0	0	1
*Streptococcus*	0	0	0	7	16	1	0	0	0	2	9	1

AMC, amoxicillin‐clavulanate; CER, ceftiofur, SI; CHL, chloramphenicol; CLI, clindamycin; ENR, enrofloxacin; ERY, erythromycin, YES; FOX, cefoxitin; GEN, gentamicin; PEN, penicillin, Cows; QD, quinupristin‐dalfopristin; SXT, trimethoprim‐sulfamethoxazole, cows; TET, tetracycline.

On an individual species level (Table 4), the highest resistance levels were found in *B*. *licheniformis* to CLI (96.6%, 29/30) and ERY (36.6%, 11/30) and the next highest resistance levels found in *B*. *subtilis* to CLI (77.7%, 7/9) and ERY (33.3%, 3/9). *Staphylococcus* was composed of seven different species (Table 5). Of these 28.5% (2/7) had resistance to both CER and ERY, and 14.2% were resistant to TET (1/7), 42.8% (3/7) were resistant to PEN and FOX (Table 5). FOX and TET resistance is indicative of methicillin resistance within these Staphylococcal species. *Escherichia* species (Table 3) comprised only *E*. *coli*, of which 3/8 (37.5%) of those isolated were resistant to AMC. The remaining 62.5% (5/8) were susceptible to the eight antimicrobials. Individual isolates within the 37.5% resistant to AMC, were also resistant to CER (12.5%), FOX (12.5%), and GEN (12.5%) (Table 6).

**Table 4:** Percentage of resistance to antimicrobials within *Bacillus* species isolated from dairy cattle with endometritis

	*B*. *licheniformis*		*B*. *subtilis*		*B*. *sonorensis*		*Brevibacillus borstenlensis*		*Bacillus* – 14spp	
*Bacillus* spp.	Overall n = 30		Overall n = 9		Overall n = 4		Overall n = 5		Overall n = 22	
Antimicrobial	No.	R%	No.	R%	No.	R%	No.	R%	No.	R%
Clindamycin	29	96.6%	7	63.6%	3	75%	3	60%	18	82%
Erythromycin	11	36.6%	3	27%	1	25%	0	0%	10	45.4%

R%, percentage of resistance within each species; N, sample size of each species; No., number of resistant isolates within each species.

**Table 5:** Percentage of resistance to 12 antimicrobials within *Staphylococcal* species isolated from dairy cattle with endometritis

*Staphylococcal* spp.	*S*. *microti*		*S*. *warneri*		*S*. *Equorum*		*S*. *Hyicus*		*S*. *hominis*		*S*. *Kloosii*		*S*. *Chromogenes*	
	Overall (n = 1)		Overall (n = 1)		Overall (n = 1)		Overall (n = 1)		Overall (n = 1)		Overall (n = 1)		Overall (n = 1)	
Antimicrobial	No.	R%	No.	R%	No.	R%	No.	R%	No.	R%	No.	R%	No.	R%
Ceftiofur	0	0%	0	0%	0	0%	1	100%	0	0%	1	100%	0	0%
Erythromycin	1	100%	0	0%	1	100%	0	0%	0	0%	0	0%	0	0%
Cefoxitin	0	0%	0	0%	0	0%	0	0%	1	100%	1	100%	1	100%
Penicillin	0	0%	1	100%	0	0%	0	0%	1	100%	1	100%	0	0%
Tetracycline	0	0%	1	100%	0	0%	0	0%	0	0%	0	0%	0	0%

R%, percentage of resistance within each species; N, sample size of each species; No., number of resistant isolates within each species.

**Table 6:** Percentage of resistance to antimicrobials within *Escherichia* species isolated from dairy cattle with endometritis

	*Escherichia coli*	
*Escherichia* spp.	Overall n = 8	
Antimicrobial	No.	R%
Amoxicillin‐clavulanate	3	**37.5%**
Ceftiofur	1	12.5%
Chloramphenicol	0	0%
Enrofloxacin	0	0%
Cefoxitin	1	12.5%
Gentamicin	1	12.5%
Trimethoprim‐sulfamethoxazole	0	0%
Tetracycline	0	0%

Value in bold indicates statistical significance at P = 0.05; R%, the percentage of resistance within each species; N, sample size of each species; No., number of resistant isolates within each species.

*Enterococcus* species constituted one isolate of *E*. *hirae*, which showed resistance to CLI and susceptibility to all remaining antimicrobials tested. *Histophilus* species constituted of two isolates of *H*. *somni* and was susceptible to all tested antimicrobials with available standards CER, CHL, ENR and GEN.

## Discussion

To our knowledge no previous studies on the uterine bacteria regarding endometritis cases have been undertaken within Australia dairy herds. Very little is known about the bacterial populations that predominate in Australia. Antimicrobial therapy is therefore being used with poor understanding of the etiological agents being treated and their susceptibility to antimicrobial therapies.

*Bacillus* spp. was the most abundant group isolated in this study. *Bacillus* spp. are gram positive endospore forming bacteria, ubiquitous in the environment and commonly isolated from dairy farms in feed, environment and milk.^26^, ^27^ *Bacillus* spp. are accepted as opportunistic uterine pathogens^22^ and are commonly isolated in studies investigating the uterine microbiome.^21^, ^28^ However, their role in disease is not fully understood.^21^ *B*. *licheniformis* has been indicated in bovine abortions,^21^ increased inflammatory mediators are produced in response to in vitro application of *Bacillus pumilis* ^29^ and *Bacillus cereus* has been isolated from necrotising placentitis causing abortion in cattle.^30^ In this study *Bacillus* spp. were isolated from all VDS scores, in both mixed populations and as singular isolates. Such a broad presence supports an opportunistic or contaminant role for these bacteria, potentially due to a high environmental load and exposure at calving. It has been demonstrated that *T*. *pyogenes*, *E*.*coli* and *F*. *necrophorum* are the key aetiological agents in endometritis.^31^, ^32^, ^33^ In this study, a low abundance of *T*. *pyogenes* and *E*. *coli* were isolated, and *F*. *necrophorum* was not isolated at all. Potentially our data may have been limited by the ability of fastidious organisms to grow by the methods used or due to the way our inclusion criteria was defined. All *T*. *pyogenes* isolates were obtained from VDS scores of 3. Our study confirmed the findings of other authors, which found *T*. *pyogenes* isolates associated with a vaginal discharge score of 3.^22^, ^34^ This finding suggests that these cattle were suffering from clinical endometritis in comparison to those cattle with a VDS of 0–1. During the first week postpartum *E*. *coli* has been the predominant bacteria observed by other authors, our study agrees with this finding due to the low numbers of *E*. *coli* isolated at 60–100 days PP.^35^ *Streptococcal* spp. were the second most isolated genre in this study, these bacteria are a common pathogen associated with endometritis in mares, specifically *Streptococcus zooepidemicus* ^36^ but have not been indicated as a primary pathogen in cattle. The *Streptococcal* spp. isolated in this study were all alpha haemolytic species with the exception of *S*. *uberis*, a known pathogen associated with mastitis.^37^ Infection in cattle with alpha haemolytic *Streptococcal* spp. has been associated with an increase in neutrophil recruitment early postpartum, and is negatively associated with infection with *T*. *pyogenes*.^38^, ^39^ Therefore, it is unlikely that the *Streptococcal* spp. isolated are causing disease and are likely commensals or contaminants. The *Staphylococcal* species isolated are considered opportunistic pathogens,^40^ with the exception is *Staphylococcus aureus* which was not isolated in this study but has been identified as a potential pathogen involved in endometritis.^40^

Considering that exposure to antimicrobials is key to the development of resistance, it is relevant to point out that some bacterial species showed resistance against antimicrobials not used in cattle in Australia and susceptibility to antimicrobials traditionally administered to Australian cattle. For example, *Bacillus* showed some resistance to clindamycin (not used in Australian cattle) and susceptibility to all antimicrobials commonly used in cattle in Australia, except for erythromycin (amoxicillin‐clavulanate, ceftiofur, penicillin and trimethoprim‐sulfamethoxazole). Similarly, *Streptococcus* showed different resistance levels against several antimicrobials not used in Australian cattle, like clindamycin, enrofloxacin, quinupristin‐dalfopristin and tetracycline. The main results of clinical importance, even in such a small sample size, are the resistance found to ENR in all species of *Streptococcus* and *Enterococcus* isolated. ENR is fluroquinolone, a reserve class antimicrobial, not labelled or licensed for food‐producing animals such as dairy cattle in Australia. As declared by the Australian Pesticides Veterinary Medicines Authority, and yet resistance was found in all species isolated in this study. ENR belong to the antimicrobial class, which is classified as a critically important antimicrobial in human medicine.^41^ Although ENR is not used in humans, there is the potential to select for cross‐resistance to antimicrobials commonly used for human therapies.^41^ Low to intermediate resistance to fluoroquinolones has been reported in veterinary streptococci but is rare.^37^ Fluoroquinolone resistance is mainly caused by selection pressure arising from the use of fluoroquinolones that cause specific mutations in the chromosomal genes known as quinolone‐resistance determining region (QRDR) of the gyrase and/or topoisomerase IV genes (gyrA, parC) that spread through horizontal transmission. Whereas the low level resistance is possible due to resistance carriage on plasmids such as qnrs. Therefore, further whole genome sequencing can be useful to understand the possible association of this antibiotics with mutation or plasmid carriage. Growing fluoroquinolone resistance has been observed in North America and in Europe, where use is allowed.^37^, ^42^ Interestingly, in this study, isolates were distributed amongst the six farms, suggesting that resistance could potentially be widely distributed amongst farms within the south‐west of Western Australia. Due to the small sample size assessed in this study, it would be prudent for further research to investigate this finding. Multidrug resistance was found in *S*. *luteinises* to CLI, ENR, QD and TMS. Resistance genes to these structurally unrelated macrolides and lincosamides, including QD, have previously been identified in *Streptococcal* spp. and attributed to the common mechanism of action of these antimicrobials.^37^ It is also noteworthy that the controls utilised in this study were to check that the MHA did not have a high thymidine content that can lead to false TMS resistance.

The *Staphylococcal* species isolated in this study was small (7/118) but showed resistance to the beta lactam families; CER, FOX, PEN, the macrolide ERY and to TET. Resistance genes in *Staphylococcal* spp. to all these antimicrobials have been identified previously and include the genes mecA, ermA, ermB and ermC, tetk/m and beta lactamase.^43^ Importantly, these resistance genes to FOX and TET are indicative of methicillin resistance within the *Staphylococcal* species isolated. Methicillin‐resistant *Staphylococcal aureus* (MRSA) was not isolated in this study but is an important pathogen associated with multidrug‐resistant infections in both humans and animals.^44^ MRSA is extremely important where human health is concerned as the transmission from livestock to producers, workers, and veterinarians occur through interaction with infected animals.^44^ *S*. *aureus* is a common pathogen associated with mastitis in dairy cattle, and transmission of methicillin resistance genes from other species could potentially lead to infections in humans.

Resistance to ERY and CLI was present in all species of *Bacillus* that were isolated. Macrolide and lincomycin resistance genes have been identified as part of the genome in *B*. *licheniformis* and are considered intrinsic but are not always expressed.^45^ In the case of CLI, intrinsic resistance has been identified but nucleotide deletions in the promotor region of this gene, can induce sensitivity to CLI.^45^ Resistance to ERY and CLI, was not only found in *B*. *licheniformis* in this study but in all species of *Bacillus* isolated, so it is likely that other *Bacillus* spp. express these same intrinsic genes and variability in resistance.

*E*. *coli* isolates had the most resistance to AMC (37.5%) but the majority were susceptible to all antimicrobials tested. CER, FOX and GEN had low levels of resistance in these isolates. Contrary findings have been identified by Brodzki et al., who reported 100% susceptibility to AMC, CER and GEN in isolates obtained from bovine endometritis samples.^19^ Chloramphenicol was the only antimicrobial with no resistance in any bacteria, but its use is not approved in Australia for production animal medicine.^41^

The limitations for this study were the sampling technique, small sample size and sample area. Despite the sterile collection of samples using a Metricheck device, there was potential contamination of the culture from purulent vaginal discharge. The use of double guarded uterine swabs is recommended. The bacteria isolated may not be representative of the dairy cattle population of Western Australia. A future larger‐scale study is needed to capture a greater geographical range and bacteria to fully evaluate the level of resistance against antimicrobials currently used in the treatment of endometritis in Western Australia. The broad sampling criteria potentially captured animals that were healthy or recovering from endometritis. To mitigate this future research should only include those animals which have a VDS >2 or using cytobrush technology to sample the uterus. The method for determining antimicrobial sensitivity relied on current CLSI standards, these are limited by the lack of standards available for some bacteria and some antimicrobials. Therefore, the use of minimum inhibitory concentrations, although more expensive, could be considered to provide an alternative to determine susceptibility in these organisms. This research, in light of its limitations is the beginning of a database of AMR in bacteria present in endometritis cases and can assist veterinarians to select the appropriate antimicrobial therapy in cases of clinical endometritis. Although resistance levels were low within the bacteria isolated, appropriate antimicrobial selection is important to prevent further resistance development.

## Conclusion

The uterine bacteria cultured in the dairy herds of south‐western Western Australia was quite diverse. The bacteria isolated varied from the accepted pathogens previously associated with clinical endometritis, but only a small sample of the population was obtained. *Bacillus*, *Streptococcus*, *Staphylococcus* species were the main aetiological agents isolated, with *T*. *pyogenes* and *E*. *coli* only rarely detected, and *F*. *necrophorum* not isolated at all. This study shows that there are low levels of resistance present to the antimicrobials tested and evidence of resistance development to important human antimicrobials, such as macrolides, cephalosporins and fluroquinolones. To support antimicrobial stewardship, the following herd management practices should be considered, improved overall nutrition, appropriate transition cow nutrition, improved hygiene when assisting dystocia cases and early assessment of at‐risk cattle post calving. The use of blanket treatment regimens with cephapirin is relied on due to its high spectrum of activity on gram positive and negative bacteria and ability of the drug to penetrate the deep layers of the uterus.

## Conflicts of interest and sources of funding

The authors declare no conflicts of interest or sources of funding for the work presented here. This work was supported by Western Dairy (grant number 17998); Dairy Australia, WA (grant number 16085) and Murdoch University.

## Appendix A

**Table A1:** Zone diameters for *Trueperella pyogenes* isolated from dairy cattle with endometritis

AMC	CER	FOX	CLI	CHL	ERY	ENR	GEN	PEN	QD	SXT	TET
0	21.0	25.1	22.2	0	0	21.7	16.0	27.7	22.9	0	16.1
23.6	24.4	28.6	26.1	23.7	26.5	22.5	16.0	21.0	25.4	0	16.4
27.0	28.8	25.1	23.2	24.6	29.0	20.8	24.2	35.1	29.0	23.7	20.2
25.1	27.6	30.9	25.3	24.8	26.1	24.1	0	27.9	32.3	0	18.6
21.5	20.1	22.6	23.0	25.7	0	24.0	17.4	32.4	28.0	0	15.5
24.7	23.0	20.2	18.5	19.6	24.3	22.7	19.0	28.1	25.9	0	10.9
22.8	22.2	19.0	17.9	22.1	22.7	21.8	18.1	28.0	26.8	0	23.1
29.5	26.7	27.0	19.8	20.1	28.6	22.5	18.0	28.6	27.3	0	16.5
23.6	21.2	29.2	24.3	24.0	30.4	22.9	21.5	32.2	27.4	0	17.6
31.2	34.0	34.8	25.2	30.9	31.9	26.1	24.0	30.5	33.1	0	19.3
28.1	24.9	30.9	23.5	20.8	29.9	20.7	21.6	23.4	20.6	27.4	12.4
28.5	20.7	24.2	24.4	21.8	24.2	21.6	19.0	23.1	18.2	0	24.5

AMC, amoxicillin‐clavulanate; CER, ceftiofur, CHL, chloramphenicol; CLI, clindamycin; ENR, enrofloxacin; ERY, erythromycin; FOX, cefoxitin; GEN, gentamicin; PEN, penicillin; QD, quinupristin‐dalfopristin; SXT, trimethoprim‐sulfamethoxazole; TET, tetracycline.

**Table B1:** Zone diameters for *Aerococcus viridians* and *Moraxella bovis* isolated from dairy cattle with endometritis

Bacteria type	AMC	CER	FOX	CLI	CHL	ERY	ENR	GEN	PEN	QD	SXT	TET
*Aerococcus viridans*	30.7	28.3	24.3	32.5	24.1	24.9	21.4	19.6	26.5	25.5	30.6	27.2
*Moraxella bovis*	32.3	21.7	31.1	9.4	32.1	20.4	29.8	22.6	26.0	19.4	26.6	22.0

AMC, amoxicillin‐clavulanate; CER, ceftiofur, CHL, chloramphenicol; CLI, clindamycin; ENR, enrofloxacin; ERY, erythromycin; FOX, cefoxitin; GEN, gentamicin; PEN, penicillin; QD, quinupristin‐dalfopristin; SXT, trimethoprim‐sulfamethoxazole; TET, tetracycline.
